# Fermented Animal Source Protein as Substitution of Fishmeal on Intestinal Microbiota, Immune-Related Cytokines and Resistance to *Vibrio mimicus* in Freshwater Crayfish (*Cherax cainii*)

**DOI:** 10.3389/fphys.2019.01635

**Published:** 2020-01-31

**Authors:** Muhammad A. B. Siddik, Ravi Fotedar, Md Reaz Chaklader, Md Javed Foysal, Ashfaqun Nahar, Janet Howieson

**Affiliations:** ^1^Department of Fisheries Biology and Genetics, Patuakhali Science and Technology University, Patuakhali, Bangladesh; ^2^School of Molecular and Life Sciences, Curtin University, Bentley, WA, Australia; ^3^Department of Marine Fisheries and Oceanography, Patuakhali Science and Technology University, Patuakhali, Bangladesh

**Keywords:** crayfish, marron, fermentation, poultry by-product, cytokines, intestinal microbiome, *Cherax cainii*

## Abstract

A feeding trial was carried out to evaluate the effects of substitution of fishmeal (FM) by dietary poultry by-product meal, fermented by *Lactobacillus casei* and *Saccharomyces cerevisiae* on growth, intestinal health, microbial composition, immune related cytokines and disease resistance of freshwater crayfish, marron (*Cherax cainii*) against *Vibrio mimicus.* Two isonitrogenous and isocaloric diets were formulated by replacing FM protein with fermented poultry by-product meal (FPBM) protein at 0% (Control) and 75% (FPBM), and fed marron for 70 days. The results indicated no significant difference (*P* > 0.05) in final body weights between two groups of marron, whilst intestinal microvilli number per fold was increased in marron fed FPBM than the control. The 16S rRNA sequences revealed an increased number of *Lactobacillus* and *Streptococcus*, and decreased number of *Aeromonas* at genus level in the distal intestine of marron fed FPBM. Marron fed FPBM showed up-regulated expression of IL-8, IL-10, and IL-17F genes in the distal intestine. Significantly (*P* < 0.05) increased lysozyme and phagocytic activity, and higher survival was found in marron fed FPBM following a bacterial challenge with *Vibrio mimicus*. Therefore, it is concluded that FPBM is beneficial to marron in terms of microbial community, immune-related cytokines and disease resistance against *V. mimicus.*

## Introduction

Aquaculture, one of the fastest growing food-producing sectors in the world, produced 80 million tonnes of edible fish valued at USD 231.6 billion in 2016, with crustacean aquaculture contributing 7.9 million tonnes with a value estimated at USD 57.1 billion (FAO, 2018). Mass production through intensive farming practices may possibly result in the prevalence of pathogens and subsequent disease infestation (Thanasaksiri et al., 2019). As a result, utilization of drugs and antibiotics to control pathogens and disease outbreaks is now common practice in aquaculture. Therefore, several issues such as the development of antibiotic resistant bacteria and the accumulation of antibiotic residues in fish tissue with the potential to be transferred to humans, has resulted in many countries banning the utilization of these drugs in aquaculture production (Pourmozaffar et al., 2019). Hence it has now become an research imperative to develop functional aqua-diets through advanced approaches like fermentation, supplementation of immune-stimulant, natural therapeutics, probiotics, and micronutrients, not only to improve the growth performance, but also, to enhance immune response and disease resistance against invading pathogens (Siddik et al., 2019b).

Microbial and yeast fermented aqua-diets have been used in fish farming for decades with much research evaluating potential on a variety of finfish (Kader et al., 2012; Siddik et al., 2019a, b) and crustaceans (Ding et al., 2015; Elshopakey et al., 2018). Previous studies have reported a range of possible functional properties in fermented products, including probiotic, antimicrobial and antioxidant properties, and the degradation of anti-nutritive compounds (Chettri and Tamang, 2014; Hill et al., 2014). Elshopakey et al. (2018) reported that dietary administration of yeast and *Lactobacillus* fermented vegetable product in the diet of kuruma shrimp, *Marsupenaeus japonicas*, elevated the innate immune response in terms of total haemocyte count, phagocytosis, bactericidal activity and increased resistance against *Vibrio parahaemolyticus*. Antimicrobial peptides derived from fermentation can also upregulate the immune and stress-related gene expression. For instance, dietary incorporation of 25% fermented soybean meal in the diet of freshwater prawn, *Macrobrachium nipponense* significantly upregulated the mRNA levels of the superoxide dismutase [Cu-Zn] gene (Ding et al., 2015).

The intestine of animals is a complex microbial ecosystem harboring a dynamic consortium of microorganisms, which can play pivotal roles in host health with multiple functions, including absorbing nutrients, improving energy production, and balancing the immune response (Hooper et al., 2002; Bates et al., 2006; Semova et al., 2012). Previous studies have found that ingestions of microbial fermented diets modulated the gut microbes of animals with the most common changes in stomach and small intestine due to fermentation reported to be an increase in the concentration of lactic acid bacteria and numbers of yeast cells (Canibe and Jensen, 2003; Missotten et al., 2015). These microbiological changes can act to protect the animals from enteropathogens (Missotten et al., 2015). Ashayerizadeh et al. (2017) reported that fermented rapeseed meal reduced the colonization of *Salmonella enterica serovar* in the gut and internal organs of broiler chicks, potentially protecting against *Salmonella* infections.

It has been reported that a omnivorous freshwater Australian crayfish, marron (*Cherax cainii*) can be successfully cultured using poultry by-product meal (PBM), feather meal, lupin meal, or meat and bone meal as the main protein source (Saputra et al., 2019). In that study, it was found that PBM as an alternative protein source to FM increased the total haemocyte count, microvillus length and susceptibility to high temperature (30°C) exposure (Saputra et al., 2019). In addition, PBM supplemented with black soldier fly (*Hermetia illucens*) meal revealed a higher bacterial activity and gene function correlated to biosynthesis of protein and energy in marron (Foysal et al., 2019a, b). However, little is known pertaining to the dietary effects of fermented PBM on the health status and intestinal microbiota of marron. Thus, the present study aimed to evaluate the effects of dietary inclusion of FPBM on the growth, immune response, intestinal microbiota and disease resistance of marron against *Vibrio mimicus*.

## Materials and Methods

### Ethics Statement

Laboratory experiment with crustacean does not require specific permits in Australia. The specimen collection site is not privately owned or protected in any way, and the study did not involve endangered or protected species. The experimental was conducted under university protocols to avoid animal suffering.

### Experimental Feeds

All feed ingredients used in this study were purchased from Specialty Feeds Pty. Ltd., Western Australia and ground into a fine powder of less than100-mesh particle size. The raw PBM was fermented by Baker’s yeast, *Saccharomyces cerevisiae* and *Lactobacillus casei* following a technique described in our earlier study (Siddik et al., 2019a). The amino acid compositions of raw and fermented poultry by-product meal (FPBM) are presented in Table 1. Two isonitrogenous (30.0% protein) and isoenergetic (19.0 MJ Kg^–1^) diets were produced with FM replaced by FPBM at a level of 0% (control) and 75%. All pre-weighed, dry ingredients were mixed and homogenized in a Hobart mixer with distilled water. The dough of the mixed ingredients was then processed with a pellet extruder to produce 3 mm pellets. The resulting pellets were air dried in the oven at 60°C for approximately 30 h and then stored at 4°C until use for feeding trials. Experimental diet ingredients and compositions are presented in Table 2.

**TABLE 1:** Amino acid composition of experimental diets (mg/g).

	PBM^†^	FPBM^‡^
Histidine	12.4	12.8
Serine	22.7	23.2
Arginine	37.4	37.6
Glycine	52.7	54.0
Aspartic acid	47.9	49.7
Glutamic acid	76.1	78.9
Threonine	22.8	23.4
Alanine	36.0	37.3
Proline	34.9	35.6
Lysine	37.1	38.6
Tyrosine	16.5	16.8
Methionine	11.5	11.8
Valine	27.3	28.4
Isoleucine	23.2	23.7
Leucine	39.9	41.0
Phenylalanine	22.2	22.7

*^†^PBM: poultry by-product meal. ^‡^FPBM: fermented poultry by-product meal.*

**TABLE 2:** Feed ingredient and nutrient composition of experimental diets (g kg^–1^).

Ingredient*	Control	FPBM
FM^†^	610.00	152.50
FPBM^‡^	0.00	446.00
Soya bean meal	100.00	100.00
Wheat flour	170.00	172.50
Wheat starch	48.00	48.00
Fish oil	42.00	52.00
CaCO_3_	0.20	0.20
Vitamin premix	2.30	2.30
Vitamin C	0.50	0.50
Cholesterol	5.00	5.00
Lecithin	10.00	10.00
Betacaine	12.00	12.00
**Nutrient composition (% dry weight basis)**		
Crude protein	29.93	29.61
Crude lipid	7.12	7.32
Gross energy (MJ kg^–1^)	18.21	18.75

*Supplied by Specialty Feeds, Perth, Australia. ^†^FM (Fishmeal): 64.0% protein, 10.76% lipid and 19.12% ash. ^‡^FPBM (Fermented poultry by-product meal): 66.98% protein, 11.70% lipid and 14.68% ash.

### Experimental System, Design, and Marron

Marron were purchased from Blue Ridge Marron Farm, Manjimup, Western Australia (34°12′21.8″S and 116°00′59.4″E) and shipped to the Curtin Aquatic Research Laboratory (CARL). Marron were acclimated in culture conditions for 1 week facilitated with constant aeration and recirculating filtered freshwater, and fed with a basal marron diet (29.0% protein, 8.0% fats, and 8.9% ash) at a rate of 1% body weight twice daily. After acclimation, 60 marron (pool initial weight 94.40 ± 2.07 g) were randomly stocked into twelve 250 L plastic cylindrical tanks (800 mm diameter, 500 mm high) where each diet was fed in six replicated group. Each tank was stocked with five marron and each marron was kept individually in a modified 750 mL plastic cage to provide shelter and avoid cannibalism during molting. All tanks were equipped with recirculating biological filtration system and an automatic submersible heater (Sonpar, Model: HA-100, China) that was set to 24°C to maintain constant temperature. Marron were fed test diets at the rate of 1% body weight for 70 days, and uneaten feed and faces were removed through siphoning every early morning. Water physico-chemical parameters, including temperature, pH and dissolved oxygen were measured daily using a portable multiparameter meter (YSI, United States), and total ammonia and nitrite were measured daily with chemical test kits (Aquarium Pharmaceuticals, Mars Fishcare North America, Inc., Chalfont, PA, United States). The measured water quality was as follows: water temperature 24.3 ± 1.8°C, pH 7.4–8.1, dissolved oxygen >5 mg L^–1^, ammonia ≤0.02 mg L^–1^ and nitrite ≤0.2 mg L^–1^. At the end of the feeding trial, marron were individually weighed to calculate final body weight (FBW), and specific growth rate (SGR) was estimated by the following formula:

$$\mathrm{Specific}\mathrm{growth}\mathrm{rate}\left(\%{\mathrm{day}}^{-1}\right)=\left[\frac{\ln\left(\mathrm{final}\,\mathrm{body}\,\mathrm{weight}\right)-\ln\left(\mathrm{pooled}\,\mathrm{initial}\,\mathrm{body}\,\mathrm{weight}\right)}{\text{days}}\right]\times 100$$

### Intestinal Micromorphology

Six marron from each control and FPBM groups (one from each tank) were sampled to dissect and extract the mid intestine for scanning electron microscopy (SEM) analysis. The SEM samples were prepared following the method described in Saputra et al. (2019). In short, the collected samples were soaked in 3% glutaraldehyde in 0.1 M cacodylate buffer overnight. The samples were then washed in cacodylate buffer and distilled water for three consecutive changes in each for 5 min, respectively. The samples were immersed in 2% OsO_4_ for 2 h followed by three washes in distilled water for 5 min, and then dehydrated using a series of 50, 75, and 95% ethanol solutions for 5 min before three final washes in 100% ethanol for 5 min. The samples were dried by washing in a series of 50%, 75% and 100% (twice) hexamethyldisilizane (HMDS) in ethanol solutions for 5 min. All processed samples were dried at room temperature and mounted on a stub using carbon tape, coated with gold and viewed under a pressure scanning electron microscope (SEM, model Phillips XL 30, FEI, Hillsboro, United States). The SEM images were analyzed to assess the length and number of microvilli by measuring and counting them on each slide using digital imaging software (Adobe Photoshop CC 2015, Adobe System Incorporated, United States).

### Intestinal Microbiome Profiling and Analysis

At the termination of the feeding trial, distal intestinal samples from three randomly selected marron from each dietary group were collected under sterile conditions. Approximately 200 mg of distal intestinal content of marron were transferred into 1.5 mL eppendorf tubes and processed for DNA extraction using DNeasy Blood and Tissue Kit (Qiagen, Crawley, United Kindom). Bacterial DNA was extracted following manufacturer’s protocols and quantified by a NanoDrop Spectrophotometer (Thermo-Fisher Scientific, Waltham, MA, United States). The extracted DNA samples were diluted to a final concentration of 30 ng/μL. PCR was performed to amplify V3-V4 region of the bacterial 16S rRNA. PCR reactions were carried out in a total volume of 50 μL of master mixture, containing Hot Start *Taq* 2X Master Mix (New England BioLabs Inc., Ipswich, MA, United States) (25 μL), template DNA (2 μL), V3-V4 sequencing primers (1 μL each), and nuclease-free water (21 μL). A total of 30 cycles of reactions were performed in a BioRad S100 Gradient Thermal Cycler (Bio-Rad Laboratories, Inc., Foster City, CA, United States). Amplified PCR products were then separated by gel electrophoresis (Bio-Rad Laboratories Inc., Hercules, CA, United States) and visualized under gel doc (FujiFilm LAS–4000 Image Analyzer, Boston Inc., Foster City, CA, United States). According to the Illumina standard protocol (Part # 15044223 Rev. B), the 16s rRNA PCR amplicon of each sample was barcoded via a secondary PCR. The V3 kit (600 cycles, Part # MS-102-3003) was used to sequence the samples up to 30,000 reads on an Illumina MiSeq platforms (Illumina Inc., San Diego, CA, United States) at the Harry Perkins Institute of Medical Research, Western Australia.

FastQC pipelines were used to check the initial quality of 16S rRNA sequences (Andrews, 2010) and QIIME (v1.6.1) pipeline was used to analyze the 16S rRNA sequence data (Kuczynski et al., 2011). The sickle program was used for quality trimming of reads and following trimming, reads with a length of <200 bp were deleted (Joshi and Fass, 2011). Merging of short reads (>100 but ≤200) was done by MefFiT pipeline (Parikh et al., 2016) and then multiple sequence alignment (MSA) was performed using PASTA algorithm (Mirarab et al., 2015). The operational taxonomic units (OTUs) were picked at 97% similarity using open reference workflow from the SILVA database (SILVA1.32 release) (Quast et al., 2013). The raw FASTQ files have been deposited in the National Centre for Biotechnology Information (NCBI) BioProject, with the accession number PRJNA521663.

Principal coordinate analysis (PCoA) was performed to visualize the clustering of samples for two different treatment groups based on related abundance (weighted). The Shannon, Simpson, and Fisher alpha diversity index were calculated in “Rstudio” using “vegan” package (Oksanen et al., 2018). Chao1 diversity index was calculated using formula S_chao1_ = S_obs_ + (n1)$\sqrt{/}\,2\,n\,2$, where S_obs_ = number of observed phyla/genus, n1 = number of singletons (phyla/genus captured once), n2 = number of doubletons (phyla/genus captured twice) (Militon et al., 2010). A Venn diagram for bacterial abundance regarding bacterial diversity at genus level was generated using FunRich (v3.1.3). The raw FASTQ files are currently available at National Centre for Biotechnology Information (NCBI) BioProject, with the accession number PRJNA521663.

### Cytokines Gene Expression Analysis

Intestinal samples from three marron from each control and FPBM groups were collected under aseptic conditions to analyse five selected genes from the cytokine family related to immunity (IL-1β, IL-8, IL-10, IL-17F, and TNF-α). The primer information for the selected genes are provided in Table 3. For RNA extraction, intestinal tissue samples were immediately stored in RNA *Later* (Sigma-Aldrich, Darmstadt, Germany) according to manufacturer’s instructions until future use. The samples were then thawed, dried, homogenized and ground into a fine powder. Total RNA from approximately 5 mg of intestinal tissue sample was extracted using RNeasy Mini Kit (Qiagen, Hilden, Germany) and extracted RNA was treated with RNase free DNase-I (Qiagen, Hilden, Germany) to remove residual DNA related contamination. Isolated RNA quality was determined in a gel electrophoresis and quantity was confirmed in a NanoDrop spectrophotometer 2000c (Thermo Fisher Scientific, United States). The cDNA library was generated from 1 μg of RNA and synthesized by Omnicript RT kit (Qiagen, Hilden, Germany) following the manufacturer’s protocols. The qRT-PCR was performed using PowerUp^TM^ Cyber Green Master Mix (Thermo Scientific, Waltham, MA, United States) with 7500 Real-Time PCR System (Applied Biosystems, Foster City, CA, United States) to measure the relative expression level of ten selected genes of the intestine tissue. The relative expression level of each gene was measured using the 2^–ΔΔCT^ method, after normalization against the β-actin reference gene (Foysal et al., 2019b).

**TABLE 3:** List of primers used in this study.

Primer	Forward sequence (5′ to 3′)	Reverse Sequence (5′ to 3′)	References
IL-1β	GTTACCTGAACATGTCGGC	AGGGTGCTGATGTTCAGCCC	Foysal et al., 2019a
IL-8	CTATTGTGGTGTTCCTGA	TCTTCACCCAGGGAGCTTC	Foysal et al., 2019b
IL-10	CAGTGCAGAAGAGTCGACTGCAAG	CGCTTGAGATCCTGAAATATA	Foysal et al., 2019b
IL-17F	GTCTCTGTCACCGTGGAC	TGGGCCTCACACAGGTACA	Foysal et al., 2019b
TNF-α	CTCAGCCATCTCCTTCTTG	TGTTCTCCTCGTTCTTCAC	Jin et al., 2018
β-actin	TTGAGCAGGAGATGGGAACCG	AGAGCCTCAGGGCAACGGAAA	Foysal et al., 2019b

### Immunological Indices

Total haemocyte count (THC) and differential haemocyte count (DHC) were estimated following the established protocol for marron (Nugroho and Fotedar, 2013). The phagocytic rate (PR) of marron was assessed according to the established protocol as described by Chen et al. (2005) and Hooper et al. (2014). Briefly, 40 μL of haemolymph was smeared on a Poly-L-Lysine glass slide (Thermofisher Scientific, United States) and incubated to make the haemolymph dry. 0.0125 g of Zymosan powder was diluted in 25 mL of sterile seawater to prepare Zymosan (Sigma-Aldrich, Z4250) working solution. Then, dried samples on the glass slide were treated with 40 μL Zymosan working solution and air dried. To incubate the cells for staining, the glass slide were placed in Giemsa solution for 20 min.

Lysozyme (LYZ) activity was determined by the turbidimetric method described in Siddik et al. (2018). Briefly, 5 mg of *Micrococus lyso* was mixed with 20 mL of PBS to prepare a bacterial suspension and stirred for 5 min. 0.3 mL of haemolymph from each marron was withdrawn using a 23-gauge needle containing a similar amount of antiquagulent, dispensed into an Eppendorf tube and mixed. 100 μL of haemolymph was pipetted and placed into a 96-well plate in duplicate. After 15 min, 100 μL of bacterial suspension was added to the wells and the mixture was incubated at 25°C. The sample was then placed in Spectrophotometer at 450 nm and absorbance was monitored every 2 min for a total of 20 min. The results were expressed as Unit mL^–1^.

### Challenge With *Vibrio mimicus*

At the end of the feeding trial, 15 average sized marron from each dietary group of control and FPBM were subjected to a bacterial challenge. For this, each dietary group had three replicates with a density of five marron per tank. The trial was conducted for 7 days with *Vibrio mimicus.* The challenge bacteria was obtained from the Department of Agriculture, Western Australia, and was grown in trypticase soy broth culture medium at 24°C for 24 h, after which the broth containing the contents were centrifuged for 15 min at 5000 × *g* and the precipitate was resuspended in phosphate-buffered saline (pH 7.2). The experimental infection was performed through the base of the fifth thoracic leg of marron by injecting with 30 μL of *V. mimicus* suspended in phosphate-buffered saline at a concentration of 3.2 × 10^6^ CFU/ml as LD_50_ (Nugroho and Fotedar, 2013). During the challenged period marron were fed once daily with the same experimental diet that was assigned before the challenge. The immunological parameters in terms of THC, DHC, PR, and LYZ in marron were analyzed before the *V. mimicus* injection and at 24 h after the injection. The survival of marron were counted at the end of the challenge trial.

### Statistical Analysis

Normality for all data were checked by Kolomogorow-Smirnov test. Two-tailed student *t*-test was applied to determine the significant difference between treatments at 5% level of significance. Kaplan–Meier, a non-parametric statistic with log rank test was used to test the probability of surviving in a given time period. The correlation analysis between the microbial community and marron immune indices was conducted using “microbiomeseq,” “phyloseq,” and “ggplot2” packages in “Rstudio.” The cycle threshold (CT) values of qRT-PCR for respective genes were analyzed using the 2^–△△CT^ method by considering reference gene (β-actin) as a control.

## Results

### Growth Performance

**FIGURE 1:**
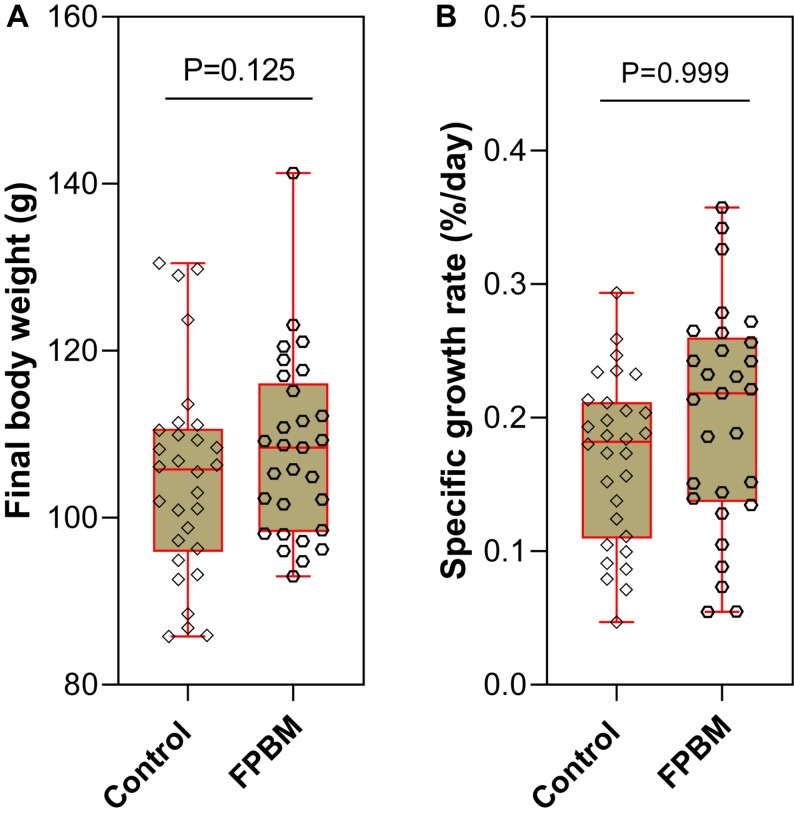
Growth performance **(A)** final body weight and **(B)** specific growth rate of marron fed control and fermented poultry by-products meal (FPBM) for 70 days. Statistical significance: unpaired two-tailed *t* test, *P* < 0.05. The line within each box represents the median. Boxplots show values obtained, whiskers indicate minimum and maximum of the dataset.

At the end of the 70 days growth trial, the mean final body weight (FBW) of marron fed FPBM (113.10 g ± 3.54) was not significantly higher than the FBW of marron fed the control diet (105.3 g ± 3.39) (*P* > 0.05). Similarly, SGR was also not significantly affected by the test diets (Figure 1).

### Intestinal Micromorphology

**FIGURE 2:**
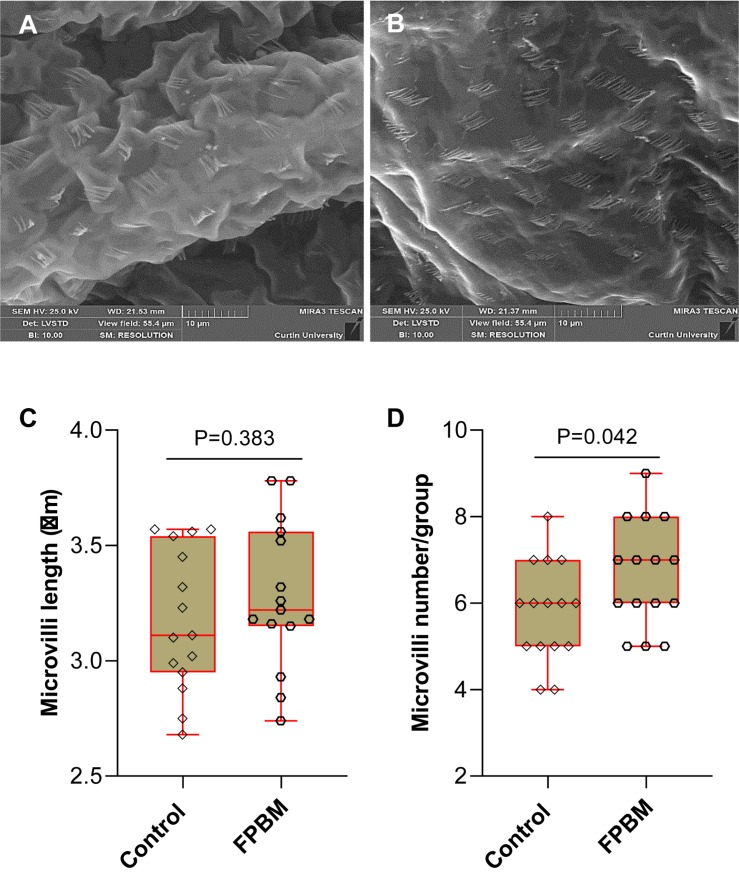
Scanning electron microscopy (SEM) of marron fed control **(A)** and FPBM diet **(B)**. Microvilli length **(C)** and microvilli number per group **(D)** were compared in the distal intestine of marron fed control and fermented poultry by-products meal (FPBM) diets. Statistical significance: unpaired two-tailed *t* test, *P* < 0.05. The line within each box represents the median. Boxplots show values obtained, whiskers indicate minimum and maximum of the dataset. 5000 × magnification; scale bars: 10 μm.

The visual images of distal intestinal surface of marron fed control and FPBM diets are shown in Figures 2A,B, respectively. The length of microvilli for marron fed FPBM was not significantly (*P* > 0.05) different than the control with the size range from 2.75 to 3.78 μm (Figure 2C). However, the microvilli number per group/fold was significantly (*P* < 0.05) higher in the FPBM than the control group with the number ranging from 4 to 9 per group in marron (Figure 2D).

### Intestinal Microbiota

**FIGURE 3:**
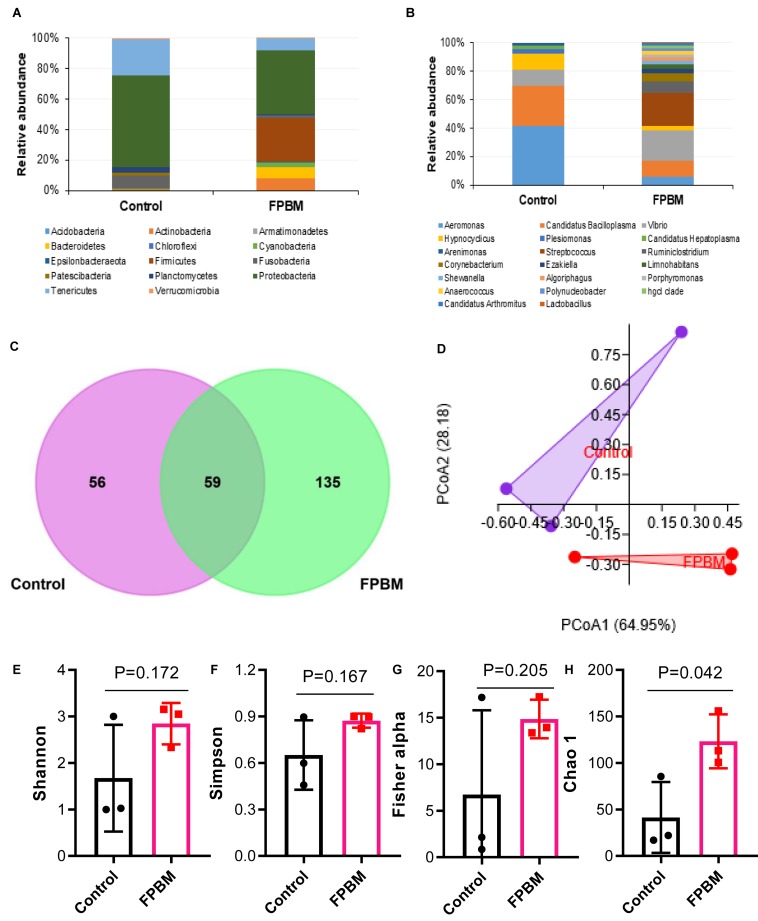
Relative abundance (%) of bacteria at **(A)** phylum, and **(B)** genus level in the distal intestine of marron fed control and FPBM diets where only bacteria with overall abundance of ≥1% were reported in the figure. Venn diagram **(C)** representing the unique and shared OTU’s between control and FPBM treated groups. Principle coordinate analysis (PCoA) **(D)** of intestinal microbial communities according to diets. Diversity indices of **(E)** Shannon, **(F)** Simpson, **(G)** Fisher alpha, and **(H)** Chao 1 between control and FPBM. *P*-value indicates significant deference in different diversity indices between the treatments as determined by two-tailed student *t* test. Data are presented as mean ± SEM, significant at *P* < 0.05.

To evaluate the effects of dietary inclusion of FPBM on marron intestinal microbiota, samples from control and FPBM treated groups were analyzed using 16S rRNA Illumina MiSeq (V3 and V4 regions). Altogether 225,266 high quality sequence reads was obtained after filtering quality and trimming the length. A total of 66,963 OTUs harbored in the intestine of marron (13904 in control and 53059 in FPBM), which mapped to 14 phyla and 119 genus at 97% similarity cut-off value. The microbial profile at the phylum and genus level with relative abundance of more than 1% are presented in Figures 3A,B. Irrespective of test diets, the dominant phyla in all tested samples were *Proteobacteria Firmicutes, Tenericutes, Fusobacteria, Actinobacteria, and Bacteroidetes*. However, FPBM significantly influenced the *Firmicutes* (28.84%), when compared to the control (0.12%) (*P* < 0.05). At the genus level, *Aeromonas* abundance significantly decreased in the FPBM treated group (4.65%) when compared to the group which received the control diet (37.77%) whereas *Lactobacillus* and *Streptococcus* were only found in FPBM group (0.15 and 18.58%) against none of these two species in the control group. PCoA based on weighted Unifrac distance revealed a clear clustering of samples according to diet, indicating the influence of diets on the intestinal microbial communities between the two groups (Figure 3C). The Venn diagram depicts that a total of 59 OTUs was shared by two groups, while the number of unique OTUs for control and FPBM groups was 56 and 135, respectively (Figure 3D). However, all bacterial diversity indices including Shannon, Simpson and Fishers alpha were not significantly influenced by diets with the exception of Chao 1, which was significantly augmented in FPBM treated groups (Figures 3E–H).

### Expression of Cytokines Gene

**FIGURE 4:**
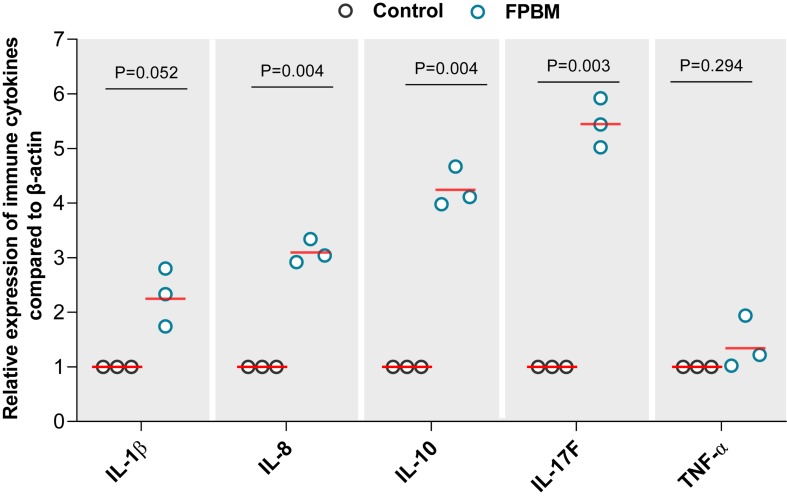
The effects of 70 days feeding with FPBM on the interleukin 1 beta (IL-1β), interleukin 8 (IL-8), interleukin 10 (IL-10), interleukin 17F (IL-17F) and tumor necrosis factor alpha (TNF-α) in the intestine of marron. *P*-value indicates significant deference between the treatments as determined by two-tailed student *t* test. *P*-value represents difference between experimental treatments. Significant difference at *P* < 0.05.

The effects of FPBM on pro-inflammatory and inflammatory cytokines mRNA expression level are presented in Figure 4. Marron fed FPBM had significantly higher IL-1β, IL-8, IL-10, and IL-17F expression when compared to the control, while TNF-α expression was not significantly impacted by the test diets.

### Immune Response

**FIGURE 5:**
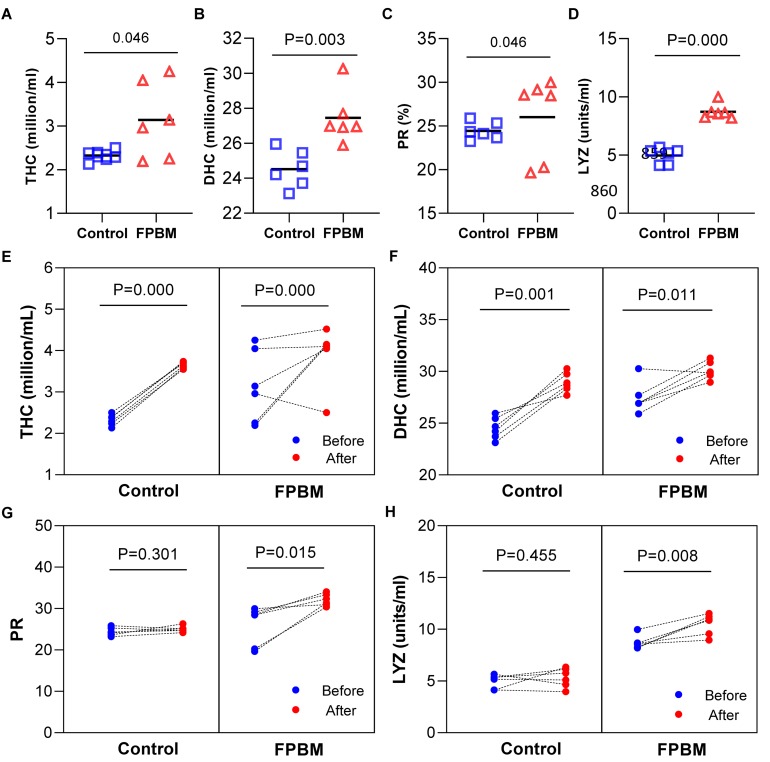
Immunological indices of marron fed control and FPBM diets. **(A)** Total haemocyte count (THC), **(B)** differential haemocyte count (DHC), **(C)** phagocytic rate (PR), and **(D)** lysozyme activity (LYZ) after growth trial for 70 days. Responses of marron before and after being challenged with *Vibrio mimicus* for 7 days **(E)** THC, **(F)** DHC, **(G)** PR, and **(H)** LYZ. *P*-value represent differences between experimental treatments. Significant difference at *P* < 0.05.

Immunological parameters of marron after 70 days of feeding trial and challenged with *V. mimicus* are shown in Figure 5. All parameters including THC (A), DHC (B) and LYZ (D), except PR (C) were significantly influenced in marron fed the FPBM diet when compared to the control. After 7 days of challenge, THC (Figure 5E) and DHC (Figure 5F) were significantly increased in the post challenge marron fed both control and FPBM diets when compared to the pre-challenge marron. In contrast, no significant difference was observed in PR (Figure 5G) between pre- and post-challenge in the control, while PR increased significantly in post challenge marron fed the FPBM. In agreement with the PR result, a similar trend was observed in LYZ (Figure 5H).

### Correlation Between Immune Indices and Bacterial Community

**FIGURE 6:**
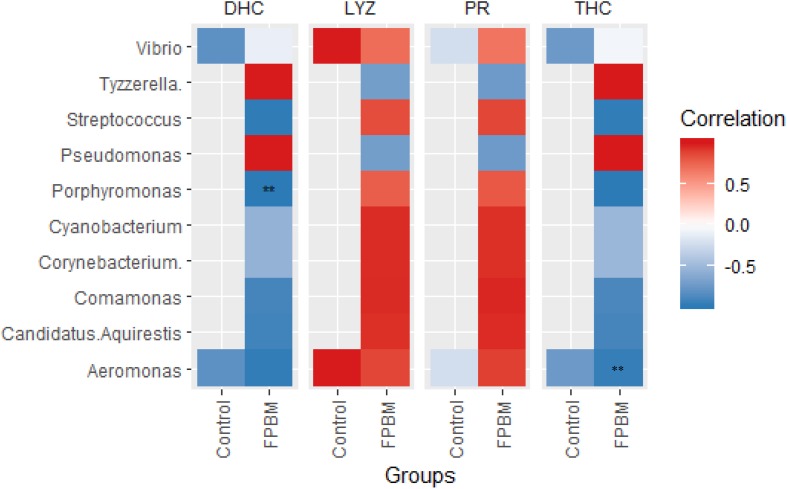
Pearson correlation plot showing the relationship among the microbial community at genus level and immune indices of marron. ** indicates significantly different at *P* < 0.05.

The Pearson’s correlation plot between microbial community abundance and immune indices of marron is presented in Figure 6, revealing that presence of *Porphyromonas* and *Aeromonas* was negatively correlated with DHC and THC, respectively, in the FPBM treated group. The LYZ and PR were not correlated with bacterial communities in the control or FPBM groups.

### *V. mimicus* Injection Challenge Test

**FIGURE 7:**
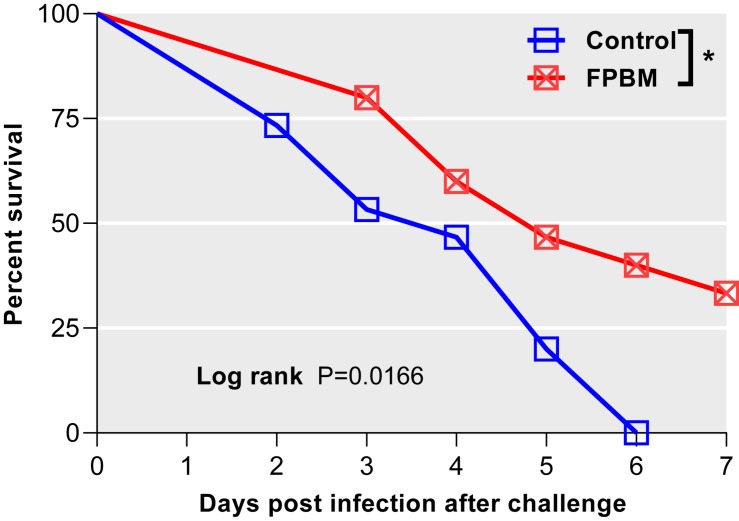
Comparison of survival of marron after *V. mimicus* challenge where *n* = 15 for each treatment. Kaplan-Meier plots with log rank test reveals that infection both in control and fermented poultry by-products meal (FPBM) started at 2 days post challenge (dpc). Marron fed FPBM showed significantly higher post challenge survival compared to control treated groups. *Indicates significant difference for survival at *P* < 0.05.

Kaplan-Meier survival curves with significant log-rank *P*-value [χ^2^ (1) = 5.74, *P* = 0.017] of marron fed control and FPBM diets are presented in Figure 7. Marron offered FPBM showed a significantly higher survival rate [χ^2^ (1) = 4.48, *P* = 0.034] following the challenge of *V. mimicus* for 7 days when compared to the control.

## Discussion

Feeding marron with FPBM had no influence on FBW and SGR in the present study. This result is in agreement with a previous study on marron fed PBM supplemented with black soldier fry insect meal and found no variations in growth performance (Foysal et al., 2019a). Past studies have also demonstrated that FM ingredients can be replaced completely by using other protein sources without negative effects on growth but compromising biochemical and immunological responses of marron (Fotedar, 2004; Saputra et al., 2019). Whilst there is little literature about the application of fermented animal protein source diets in crustacean aquaculture, there are some studies with fermented plant source diets. Ding et al., 2015 suggested that inclusion of 25% fermented soy bean meal improved the FBW of freshwater prawn, *Macrobrachium nipponense* and that further increasing the inclusion levels up to 100% did not affect the growth performance. A study on white shrimp, *Litopenaeus vannamei* fed 25% fermented soybean meal for 12 weeks found no difference in growth performance when compared to the shrimp fed the FM based control (Lin and Mui, 2017). The discrepancies amongst the findings of these studies might be attributed to a number of factors such as the raw material, fermentation conditions including the specific types of microorganisms, temperature and feed to water ratio (Canibe and Jensen, 2012; Missotten et al., 2015) and substrate quality and quantity including compositional aspects of carbohydrates, fibers, protein, amino acids and vitamins (Niba et al., 2009). As an example, the crude protein content of soybean meal was increased after fermented with *Bacillus subtilisor* and *Aspergillus oryzae* (Teng et al., 2012), whereas, the content of protein and lipid was unchanged in soybean meal fermented by *Bacillus* spp. (Yamamoto et al., 2004).

It is generally assumed that longer intestinal microvillus size and density are associated with higher absorptive efficiency, leading to better nutrient utilization and improved immune function. Dimitroglou et al. (2009) stated that good intestinal health is of great importance in improving the health status of the mucosal epithelium as well as increasing the ability to prevent invading pathogens. In the present study, higher numbers of microvilli per intestinal fold were found in marron fed with FPBM. This may be due to the presence of yeast and bacteria in FPBM which may act as probiotics and could be responsible for improved nutrient absorption and utilization leading to an enhanced immune response. Lin and Mui (2017) claimed that soybean meal fermented with *Lactobacillus* spp. prevented the suppression of immune function triggered by soybean meal in white shrimp, *Litopenaeus vannamei.* However, the results of our previous research indicated that replacing FM with non-fermented PBM leads to no significant variation in microvilli number in the midgut of marron (Saputra et al., 2019).

Recently, manipulation of gut microbiota of farmed aquatic animals through dietary modulation an emerging area for aquaculture advancement. Gut microbiota play an important role in crustaceans by participating in a variety of physiological functions including feed digestion, reduction of the prevalence of pathogenic bacteria and/or synthesis of the amino acids, vitamins and trace elements (Merrifield and Ringø, 2014; Mente et al., 2016; Gao et al., 2019). A limited number of studies have been conducted on characterization of the gut microbiota of crustaceans and hence, the present study employed 16sRNA Illumina sequencing, a modern technique, for the first time to investigate the intestinal microbiota of marron post feeding with FPBM. The results showed that regardless of the diet, *Actinobacteria, Bacteroidetes, Proteobacteria, Tenericutes, Firmicutes and Fusobacteria* were the predominant phylum in the intestine of marron, which is in line with previous studies in other crustaceans, such as *Litopenaeus vannamei* (Xiong et al., 2015; Zhang et al., 2016) and *Peneous monodon* (Rungrassamee et al., 2013). However, in the present study, the abundance of *Firmicutes* (including *Lactobacillus* spp. and *Streptococcus* spp.) significantly increased in the intestine of marron fed FPBM. Similarly, Firmicutes phylum, namely under the genera of lactic acid bacteria (LAB), such as *Lactobacillus, Lactococcus, and Pediococcus* were enriched significantly in the intestinal content of Atlantic Salmon, *Salmo salar* fed fermented soybean meal in comparison with fish fed either a non-fermented or FM-based diet (Catalán et al., 2018). Many strains of *Firmicutes* are potent probiotics due to their beneficial effects on metabolism, digestion (by releasing different enzymes), adjustment of the intestinal environment and regulation of intestinal mucosal immunity of aquatic animals (Smriga et al., 2010). Fermented products also contain a diverse group of prebiotic compounds attracting and stimulating the growth of probiotics in terms of LAB and glucans (Swain et al., 2014). In the present study, *Aeromonas* was decreased in the group fed with the FPBM based diet when compared with the group fed the control diet. *Aeromonas* is a pathogenic group of bacteria that causes infection in fish and crustaceans (Birkbeck and Ringø, 2005; Ringo et al., 2007; Chen et al., 2018). LAB used in the present study can produce lactic acid which may reduce the pH of the intestinal content, resulting in the inhibition of the proliferation of pathogenic bacteria (Parvez et al., 2006). Pearson’s correlation revealed the negative association between DHC and *Porphyromonas* in the FPBM treated group. Although the effect of *Porphyromonas* in marron is not well understood, this pathogenic bacteria genus in humans causes chronic inflammatory disease (Mysak et al., 2014). It can be suggested that the proliferation of beneficial bacteria and reduction of the prevalence of pathogenic bacteria in the FPBM diet, as shown in this study demonstrates the potential beneficial effects of fermentation bacteria and products on the gut health of marron.

Many secondary metabolites ranging from antibiotics to peptides have been produced during the process of fermentation (Hayes and García-Vaquero, 2016), which are very much effective at elevating innate and adaptive immunity (Tsai et al., 2012). Oral administration of *Lactobacillus sakei* K101 obtained from fermented dairy product and *Lactobacillus plantarum* K55-5 obtained from fermented vegetable significantly increased cytokines such as L-10, IL-12, IFN-γ, and TNF-α in mouse spleen and blood (Lee et al., 2016). Elshopakey et al. (2018) reported an elevated level of immune related cytokines encompassing antimicrobial peptides (AMPs) and toll-like receptors (TLR) in the intestine of kuruma shrimp, *Marsupenaeus japonicas* fed 5.5 and 55 g fermented vegetable product (FVP). Similarly in the present study, pro-inflammatory cytokines including TNF-α, IL-1β, IL-8, and IL-17F demonstrated higher gene expression in the intestine of marron fed FPBM. These cytokines regulate many biological activities including immune response and maintenance of homeostasis of the immune system, bactericidal activities and lysozyme synthesis (Giri et al., 2015), and enhancement of host defense mechanism against bacteria and fungi (Moulton, 2016). IL-10, an important anti-inflammatory having both immunosuppressive and immunostimulatory properties, can enhance B-cell mediated functions, proliferation and antibody production (Deng and Tsao, 2013). IL-10 responses vary with different stages of inflammation, indicative of its major role in recovering from inflammation (Foysal et al., 2019b). For instance, it has been reported that the expression level of anti-inflammatory cytokines increase aligned with overexpression of pro-inflammatory genes to neutralize inflammation (Foysal et al., 2019a). In this study, the expression level of IL-10 was significantly higher in marron fed FPBM than in the group fed the control diet.

In crustacean haemocytes, antimicrobial effector cells play an important role in regulating physiological and immunological functions by acting in both adoption of the cellular immunity and supply of the humoral immunity (Liu et al., 2009; Hong et al., 2017; Saputra et al., 2019). The THC and DHC in the circulatory system of crustaceans may be easily affected by intrinsic factors and extrinsic factors including heavy metals, pathogens and pesticides (Cheng and Chen, 2000; Yao et al., 2008). Elevation of these two haemocytes parameters may induce immune competence against environmental stress (Burgos-Aceves et al., 2018; Nedaei et al., 2019) and increase resistant in response to opportunistic pathogens (Cheng and Chen, 2001; Pagano et al., 2017). In the present study, THC and DHC increased significantly in marron fed FPBM when compared to the control after 70 days and were also elevated in both control and FPBM treated groups after challenge with *V. mim*icus. These findings are in agreement with Elshopakey et al. (2018), who reported elevated level of THC in kuruma shrimp, *Marsupenaeus japonicas* when fed with fermented vegetable product.

Lysozyme, an important component of innate immune system and antimicrobial enzyme is present in phagocytic cells, and may act as an opsonin by lysing the peptidoglycans in Gram-positive bacterial cell walls (Nilojan et al., 2017). Many studies have reported elevated levels of lysozyme expression following bacterial infection (Tyagi et al., 2007; Yao et al., 2008). In this study, the lysozyme levels of marron that received FPBM were significantly elevated following 7 days challenged with *V. mimicus* when compared to levels before challenge. Similar to the LYZ result, post challenge marron that was fed FPBM revealed significantly higher PR than the pre challenge groups. In agreement with some previous studies (Kumar et al., 2014; Elshopakey et al., 2018), the present results proved the immunomodulatory effects of FPBM on marron by elevating the level of THC, DHC, PR, and LYZ.

Enhancement of disease resistance through the modulation of nutrition has recently become an aquaculture research priority as a strategy to increase immune response and therefore to reduce the dependence on chemotherapeutics (Pohlenz and Gatlin, 2014; Elshopakey et al., 2018). Fermentation has been reported to release bioactive peptides by the action of proteolytic enzymes produced by fermented microorganisms and these peptides have been attributed to have therapeutic benefits including immunomodulatory (Lin and Mui, 2017), antioxidant (Sanjukta et al., 2015) and antimicrobial activity (Eom et al., 2014). Antimicrobial peptides (AMPs) derived from fermentation have been considered as one of the major humoral immune effectors, which can act against both gram-positive and gram-negative bacteria, viruses, fungi and protozoans (Brown and Hancock, 2006). Three antimicrobial peptides derived from *Bacillus subtilis* E20-fermented soybean meal were shown to have antimicrobial activities against *Vibrio* infection in shrimp, while no antimicrobial activities were found in un-fermented soybean meal fed shrimp (Cheng et al., 2017). In this study, a significantly higher survival rate following *V. mimicus* challenge was observed in marron fed FPBM than the marron offered the control diet. Similarly, fermented soybean product in the diet of white leg shrimp, *Penaeus vannamei* significantly increased the survival rate against *Vibrio harveyi* when compared with control shrimp given diet without these product (Yatip et al., 2018). However, mortality of freshwater prawn, *Macrobrachium nipponense* against *Aeromonas hydrophila* was not significantly different between the control and diets with up to 50% of fermented soybean meal although it increased significantly in 75 and 100% FSM treated groups (Ding et al., 2015).

## Conclusion

The results demonstrated that FPBM was immunologically favorable to marron, indicated by the elevated immune response and immune related cytokines, as well as increased numbers of beneficial microbiota. The abundance of Firmicutes bacterial communities suggest that positive effects on the gut microbial composition is possible to obtain through the manipulation of aqua-diets, and therefore, on the physiological condition of the host. Dietary inclusion of FPBM also significantly enhanced the disease resistance against *V. mimicus*, as well as enhancing the immune response in post challenge marron, indicating an immunomodulating competency of FPBM. Further studies are required to explain the mode of action of fermented product addition on intestinal microbiota and disease resistance, which may lead to the development of a novel functional feed for marron and its sustainable aquaculture production.

## Data Availability Statement

The datasets in this study can be found in the BioProject database, https://www.ncbi.nlm.nih.gov/bioproject/PRJNA521663 (accession: PRJNA521663).

## Author Contributions

MS and RF were involved in designing the experiment and proofreading the manuscript. MS and MC were involved in conducting the experiment, analyzed the data, and wrote the manuscript. MF was involved in all *in vitro* and *in vivo* analyses of the study. AN helped in innate immune response and electron microscopy analysis. JH helped in the experimental design and proofreading the manuscript.

## Conflict of Interest

The authors declare that the research was conducted in the absence of any commercial or financial relationships that could be construed as a potential conflict of interest.
